# Wavelength- and angle-multiplexed full-color 3D metasurface hologram

**DOI:** 10.1515/nanoph-2025-0504

**Published:** 2025-12-09

**Authors:** Tetsuhito Omori, Kentaro Iwami

**Affiliations:** Department of Advanced Interdisciplinary Science, 13125 Tokyo University of Agriculture and Technology, Koganei, Tokyo 184–8588, Japan; Department of Advanced Interdisciplinary Science, and Department of Mechanical Systems Engineering, Tokyo University of Agriculture and Technology, Koganei, Tokyo 184–8588, Japan

**Keywords:** metasurface, holography, wavelength multiplex, angle multiplex, silicon nitride

## Abstract

Metasurface holography is a promising technology for next-generation 3D displays, however, conventional approaches for full-colorization have faced challenges. Wavelength multiplexing based on spatial segmentation/interleaving inevitably reduces pixel density, while techniques reliant on the Pancharatnam–Berry (PB) phase are inherently polarization-dependent and have a theoretical efficiency limit of 50 %. In this work, we propose and experimentally demonstrate a design strategy that overcomes these limitations. The core of our approach is a single, polarization-independent meta-atom, realized with cross-shaped nanopillars made of silicon nitride (SiN), which enables the simultaneous and independent phase control over the three primary colors required for faithful 3D image reconstruction. This single-unit strategy surpasses the pixel density limitations of wavelength multiplexing. Furthermore, we combine this innovation with crosstalk elimination via spatial division of target 3D images and precise angle correction to ensure high-fidelity, superimposed reconstruction. Experimentally, we have successfully reconstructed high-definition, noise-free 3D full-color holograms. Our work resolves the critical limitations of pixel density and polarization dependence in metasurface holography, providing a robust pathway toward practical, high-performance holographic displays.

## 1 Introduction

Holography is an ideal three-dimensional (3D) display technology that completely records and reconstructs the wavefront of light, with promising applications ranging from augmented/virtual reality (AR/VR) and security to optical manipulation [1], [2], [3]. Computer-generated holography (CGH) has revolutionized this field due to its advantages, such as eliminating the need for filming and enabling the projection of non-existent objects [4], [5]. However, conventional dynamic holographic devices, which integrate CGH with spatial light modulators (SLMs), have faced inherent challenges such as a limited viewing angle due to large pixel sizes [6], [7].

Recently, metasurfaces – planar optical elements composed of integrated subwavelength structures (meta-atoms) – have garnered significant attention as a technology to solve these issues [8], [9]. Metasurfaces can mimic the wavefront of light with arrayed meta-atoms with subwavelength pixel pitch, making it possible to suppress unwanted light in principle while achieving both a wide viewing angle and high definition. Due to these characteristics, their application to high-performance optical elements such as lenses [10], [11], [12], [13], [14], [15], [16], [17], [18], wavelength plates [19], [20], prisms [21], [22], polarizers [23], [24], orbital angular momentum (OAM) devices [25], [26], [27], [28], [29], [30], [31], AR [32], [33], and multifunctional devices [34].

As well as these applications, many research efforts have been dedicated to metasurface holography in the decade [35], [36], [37], [38]. To increase the information capacity of a single metasurface, multiplexing technology, which expresses independent information channels with various physical degrees of freedom of light, has been actively studied. For example, projection of a full-color 2D/3D image requires multiplexing across the three fundamental RGB wavelengths. Similarly, playing a movie requires multiplexing multiple frames in the time domain. To achieve this, various degrees of freedom, for example spatial [39], [40], polarization [41], [42], [43], [44], angle of incidence [45], [46], [47], [48], [49], propagation direction [50], inverse design [51], and OAM [52], [53] have been utilized to multiplex information whether wavelength or time.

Among these, polarization multiplexing using two orthogonal components offers low crosstalk and high signal-to-noise ratio. However, they only support two channels, making them unsuitable for full-color applications requiring three channels. Furthermore, since they utilize only specific polarizations, efficiency is halved.

Other representative method is spatial multiplexing including the segment type, which spatially divides regions for each RGB color [39], [54], [55], and the interleave type, which mixes meta-atoms for each color within a unit cell [40]. They are straightforward approaches to achieve full-color metasurface holography. However, these approaches lead to a reduction in the viewing angle due to increased pitch size, as well as a decrease in resolution and increased processing difficulty due to the enlargement of the metasurface.

Yamaguchi et al. recently proposed wavelength-multiplexing method that simultaneously implements phase delay for three wavelengths using a single waveguide-type meta-atom [56]. This method achieved both full-color reproduction through three-wavelength coaxial illumination and animation through a cinematographic approach. However, to avoid crosstalk images, it required large black padding areas, leading to increased metasurface size and reduced resolution.

In this study, we propose an angle- and wavelength-multiplexed metasurface hologram that overcomes these limitations, and experimentally demonstrate high-quality full-color holographic 3D image reconstruction that large enough to be observed with naked-eyes. Our approach is founded on a single, high-performance, cross-shaped meta-atom made of low loss silicon nitride (SiN) waveguide-type meta-atom. By simultaneously and independently controlling the phase of all three RGB wavelengths, this single-unit strategy overcomes the pixel density limitations of coaxial wavelength multiplexing, while its isotropic geometry ensures complete polarization-independent operation. The successful reconstruction of high-quality full-color 3D images is ensured by combining these innovations with deterministic crosstalk elimination via spatial division of target 3D images and precise angle correction using Rodrigues’ formula. Overcoming these technical challenges opens the way for more advanced applications in future dynamic holographic displays [57], [58].

## 2 Principles

**Figure 1:**
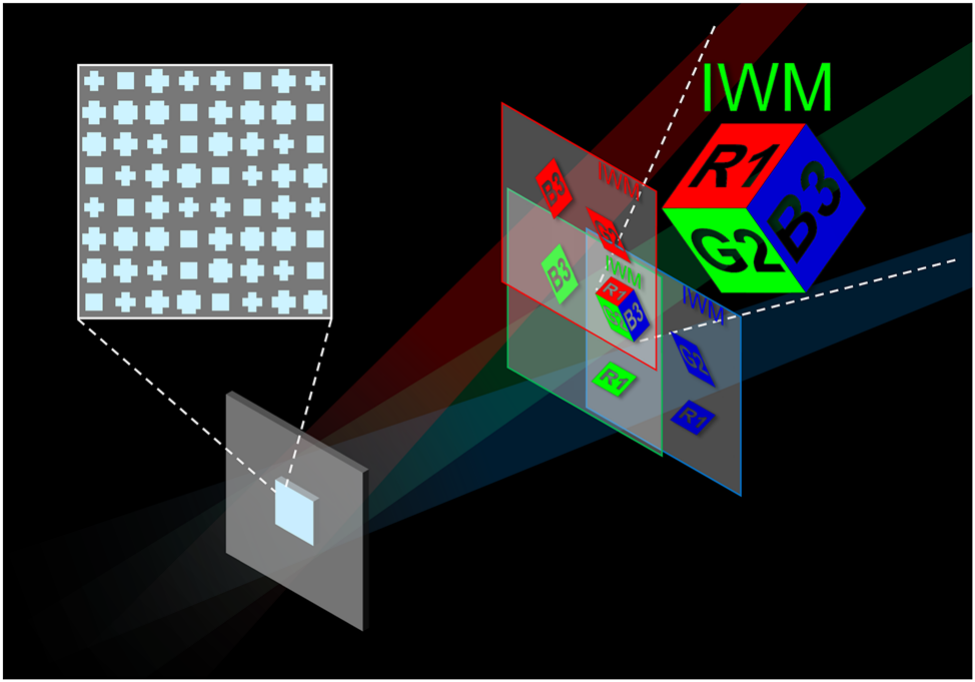
Schematic of the proposed full-color 3D metasurface hologram. A single, polarization-independent meta-atom simultaneously controls three primary wavelengths (RGB). Oblique illumination spatially superimposes the reconstructed 3D images into a single full-color 3D image while avoiding crosstalk.

**Figure 2:**
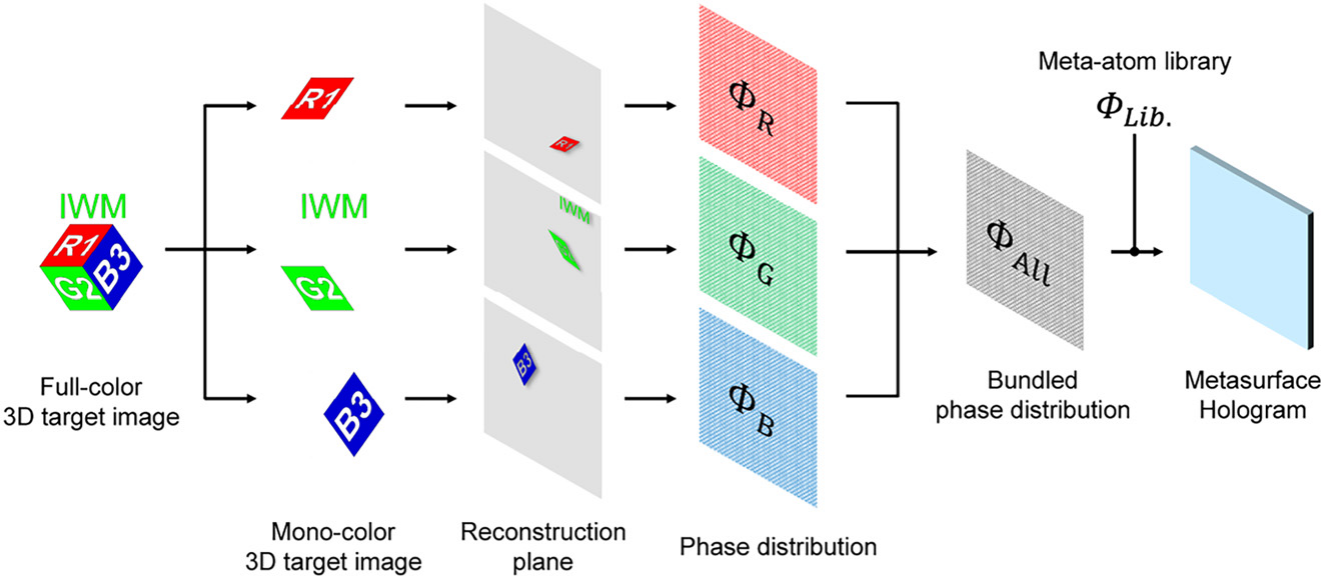
Principle and workflow of the proposed full-color 3D metasurface hologram.

The concept of our method is schematically illustrated in Figure 1. Our method is founded upon two core principles: the independent phase control of three wavelengths by a single meta-atom, and the deterministic separation of crosstalk by spatially dividing the reconstructed 3D images, while changing incident angles for each wavelength to superimpose them at the same location. The overall design workflow is shown in Figure 2.

**Figure 3:**
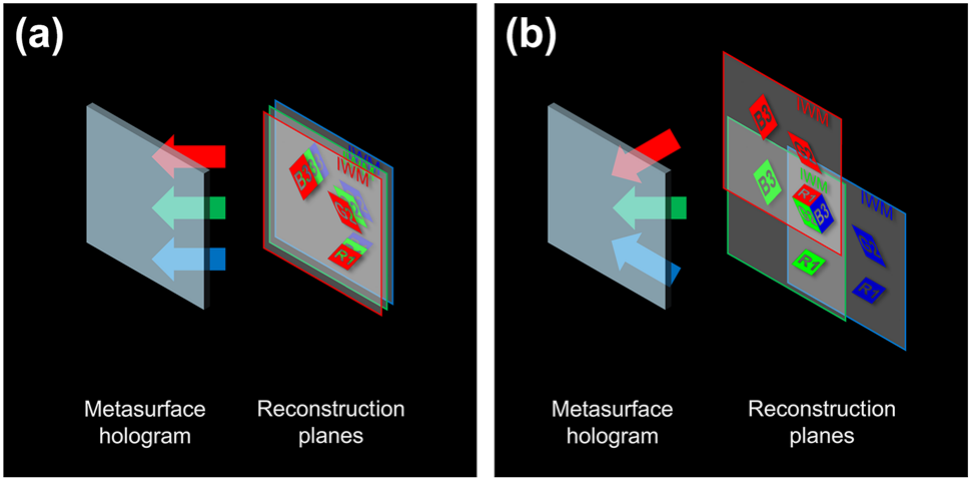
Concept of superimposing for the reconstructed 3D images. (a) Under normal incidence. (b) Under oblique incidence, the 3D images are superimposed to form a single, full-color 3D image, avoiding overlap with crosstalk images.

The design process begins with defining the target 3D image on the reconstruction plane. First, a desired 3D Full-color target object is decomposed into three mono-color channels for the primary colors (RGB). Next, to prevent crosstalk, these target 3D images are arranged in non-overlapping regions on the reconstruction plane, as illustrated in Figure 3. Here, if we could completely reproduce phase distributions for each color independently, only the wanted objects could be reconstructed as shown in Figure 2. However, in practice, due to the limited degrees of freedom of meta-atoms, perfect phase control for all three colors is not achievable. Thus, unwanted crosstalk 3D images generated by unintentional diffraction – for instance, green and blue diffraction light from the red design – inevitably occur as shown in Figure 3(a). These unwanted images should be physically displaced from the desired signal regions, ensuring they do not overlap with the target 3D image, as shown in Figure 3(b). At this time, as detailed in the Design section, the reconstructed 3D image is rotated on a spherical surface centered on the hologram according to the incident angle of the illuminating light. Thus, simply translating the target image causes distortion and blur, preventing high-quality full-color reconstruction. Therefore, the angle of the target 3D image is corrected using Rodrigues’ rotation formula, as described later in the Design section.

**Figure 4:**
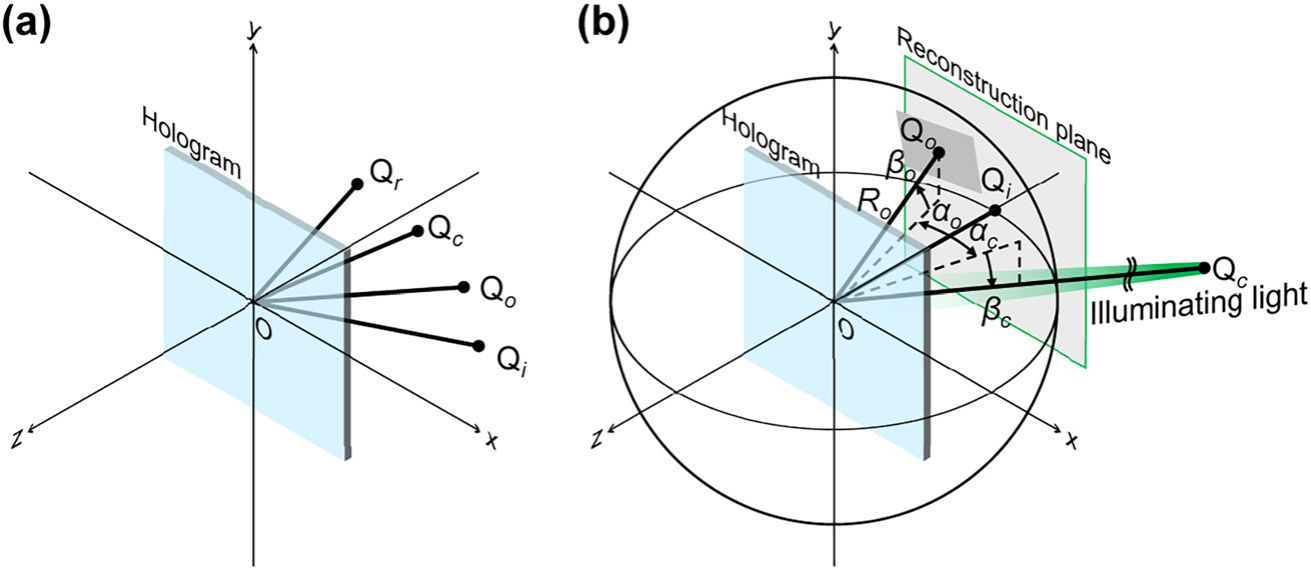
Imaging in holography. (a) Overview of light waves. Point object Q_*o*_, a reference point light source Q_*r*_, a illumination light source Q_*c*_, and a reconstructed image Q_*i*_. (b) Imaging in this study. The reconstructed 3D image is rotated on a spherical surface centered on the hologram.

To achieve this concept, we consider the oblique incidence based on Figure 4. As shown in Figure 4(a), we consider a point object Q_*o*_, a reference point light source Q_*r*_, an illumination light source Q_*c*_, and a reconstructed image Q_*i*_. The imaging formula between them are expressed by the following equations [59]:

(1) $$\frac{1}{\lambda_{c}}\left(\frac{1}{R_{i}}-\frac{1}{R_{c}}\right)=\pm \frac{1}{m^{2}\lambda_{r}}\left(\frac{1}{R_{o}}-\frac{1}{R_{r}}\right)$$

(2) $$\frac{\sin\alpha_{i}-\sin\alpha_{c}}{\lambda_{c}}=\pm \frac{\sin\alpha_{o}-\sin\alpha_{r}}{m\lambda_{r}}$$

(3) $$\begin{array}{ll} & \frac{\cos\alpha_{i}\sin\beta_{i}-\cos\alpha_{c}\sin\beta_{c}}{\lambda_{c}}\\ & \;=\pm \frac{\cos\alpha_{o}\sin\beta_{o}-\cos\alpha_{r}\sin\beta_{r}}{\lambda_{r}} \end{array}$$

where *λ* is the wavelength, and (*R*, *α*, *β*) are the spherical coordinates, as illustrated in Figure 4(b). The subscripts *o*, *r*, *c*, and *i* denote the object, reference, illumination, and reconstructed image waves, respectively, and *m* is the magnification. For our system, we assume the reference beam is a plane wave normally incident to the hologram plane (*α*_*r*_ = *β*_*r*_ = 0, *R*_*r*_ = *∞*), the reconstruction is performed with a plane wave (*R*_*c*_ = *∞*). With unitary magnification (*m* = 1) and identical recording and reconstruction wavelengths (*λ*_*c*_ = *λ*_*r*_), the imaging equations (Eqs. (1)–(3)) are simplified to:

(4) $$R_{i}=\pm R_{o}$$

(5) $$\sin\alpha_{i}=\sin\alpha_{c}\pm \sin\alpha_{o}$$

(6) $$\cos\alpha_{i}\sin\beta_{i}=\cos\alpha_{c}\sin\beta_{c}\pm \cos\alpha_{o}\sin\beta_{o}$$

These equations indicate that the reconstructed 3D image is rotated on a spherical surface centered on the hologram according to the incident angle of the illuminating light as shown in Figure 4(b). If we align the reconstructed image on the *z* − axis (*α*_*i*_ = *β*_*i*_ = 0), we obtain one solution that (*α*_*c*_, *β*_*c*_) = (−*α*_*o*_, − *β*_*o*_). Thus, if the object angle (*α*_*o*_, *β*_*o*_) is small and the difference between the spherical surface and reconstruction plane is negligible, we can approximately align the object on there. Furthermore, by selecting the appropriate incident angles (*α*_*c*_, *β*_*c*_) for each color, the spatially divided object waves are reconstructed at the same location on the *z* − axis, forming a full-color target 3D image, as shown in Figure 3.

Subsequently, the ideal complex amplitude distribution on the hologram plane is calculated for each spatially divided target 3D image using the angular spectrum method. From this, we extract the required phase distribution for each color *C* ∈ {*R*, *G*, *B*}, denoted as Φ_*C*_, which is a matrix of phase values *ϕ*_*C*,*ij*_ for each pixel at position (*i*, *j*):

(7) $$\Phi_{C}=\left(\phi_{C,ij}\right),\;\phi_{C,ij}\in \left[0,2\pi \right]$$

To realize a full-color hologram with a single metasurface, each meta-atom must simultaneously satisfy the phase requirements for all three colors. We therefore define the bundled phase distribution for the entire metasurface, Φ_All_, which is composed of a target phase vector ***ϕ***_All*,ij*_ for each pixel at position (*i*, *j*) as follows:

(8) $$\Phi_{\mathrm{All}}=\left(\phi_{\mathrm{All}\mathrm{,ij}}\right),\;\phi_{\mathrm{All}\mathrm{,ij}}=\left(\begin{array}{c} \phi_{R,ij}\\ \phi_{G,ij}\\ \phi_{B,ij} \end{array}\right)$$

To physically implement this target phase vector, we designed a library Φ_Lib*.*_ consisting of *n* = 189 meta-atoms through the electromagnetic field analysis as detailed later. We adopted waveguide-type meta-atoms with cross-shaped geometries made of silicon nitride. Each entry in the library corresponds to a specific meta-atom geometry and its resulting phase vector ***ϕ***_Lib*.,n*_:

(9) $$\phi_{\mathrm{Lib}\mathrm{.,n}}=\left(\begin{array}{c} \phi_{R,n}\\ \phi_{G,n}\\ \phi_{B,n} \end{array}\right)\;\left(\in \Phi_{\mathrm{Lib.}}\right)$$

For each pixel (*i*, *j*) on the metasurface, we then select the optimal meta-atom by finding the entry in Φ_Lib._ that minimizes the Euclidean distance to the target phase vector ***ϕ***_All*,ij*_. This matching process determines the final selected phase vector, ***ϕ***_Lib*.,ij*_, for the meta-atom array:

(10) $$\phi_{\mathrm{Lib}\mathrm{.,ij}}=\underset{\phi_{\mathrm{Lib}\mathrm{.,n}}\in \Phi_{\mathrm{Lib.}}}{\mathrm{arg min}}\|\phi_{\mathrm{All}\mathrm{,ij}}-\phi_{\mathrm{Lib}\mathrm{.,n}}\|$$

## 3 Design

**Figure 5:**
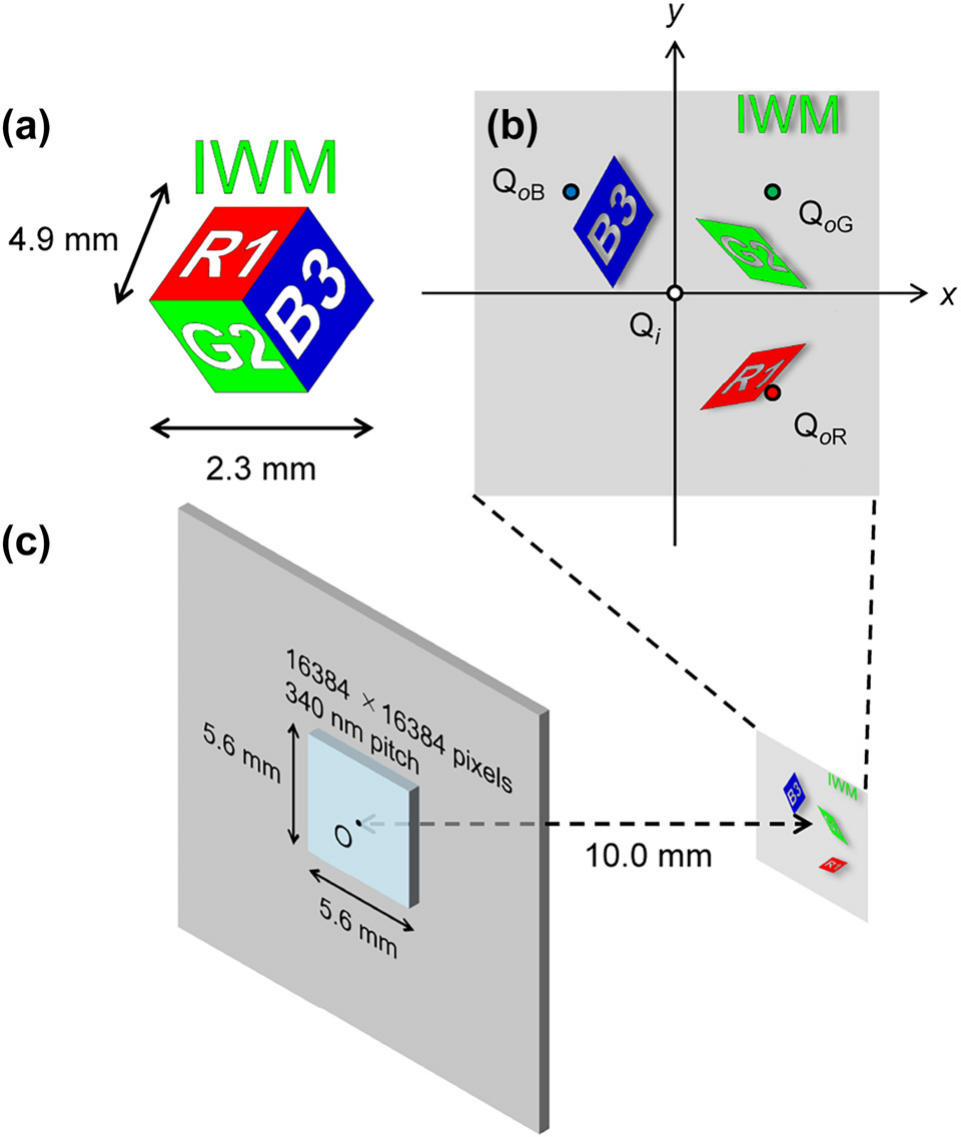
Hologram design parameters. (a) The target 3D image with an outer diameter of 2.3 mm and a depth gap between the foreground cube and the background text of 4.9 mm. (b) Spatial division of the angle-corrected monochromatic 3D images on the reconstruction plane, with coordinates defined from the origin O at the hologram’s center. Superimposing position Q_*i*_ = (0, 0, − 10.0), object positions Q_*o*R_ = (1.4, − 1.4, − 10.0), Q_*o*G_ = (1.4, 1.4, − 10.0), Q_*o*B_ = (−1.4, 1.4, − 10.0) Scale: mm. (c) The hologram has a size of 5.6 mm × 5.6 mm (16,384 × 16,384 pixels) with a 340 nm pitch, and the reconstruction distance is 10 mm.

The target 3D model, as illustrated in Figure 5(a), consists of a cube and the text ”IWM”. The surface data of these models are prepared as wrl files for each wavelength channel, using 3-D computer aided design (CAD) software. These surface files are loaded into WaveField Tools (WFT) 3.9*β* (Prof. K. Matsushima, Kansai University, Japan), a wave optics calculation toolkit, as a polygon light sources.

As shown in Figure 4(b), the polygon light sources are rotated on a spherical surface centered on the hologram according to the incident angle of the illuminating light, using WFT. To compensate for the rotational shift from oblique illumination, we apply an inverse rotation to the target 3D image’s coordinates using Rodrigues’ rotation formula:

(11) $$v^{\prime }=v\cos\theta +\left(n\times v\right)\sin\theta +n\left(n\cdot v\right)\left(1-\cos\theta \right)$$

where **v′** is the rotated vector, **v** is the original vector, **n** is the unit vector of the rotation axis, and *θ* is the rotation angle. In this study, as illustrated in Figure 5(b), we define the desired superimposing position as a point Q_*i*_ and the object positions for each wavelength as Q_*o*R_, Q_*o*G_ and Q_*o*B_. With the origin O at the center of the hologram, the angle of the target 3D image for each wavelength is then corrected using Rodrigues’ rotation formula by setting the vector **v′** as the respective vectors $\vec{{OQ}_{oR}}$ , $\vec{{OQ}_{oG}}$ , and $\vec{{OQ}_{oB}}$ and the vector **v** as the vector $\vec{{OQ}_{i}}$ .

After applying Rodrigues rotation, the object light wave was stored in the frame by the polygon-based method [60], [61] on the reconstruction plane, and propagation calculation was performed using the angular spectrum method on WFT to obtain the phase distribution on the hologram surface. As specified in Figure 5(c), the distance from the hologram plane to the reconstruction plane was set to 10 mm. The hologram size was 5.6 mm × 5.6 mm (16,384 × 16,384 pixels) with a 340 nm pitch. Subsequently, based on the phase distribution on the hologram surface for each wavelength, the optimal meta-atom was selected from the meta-atom library, and the metasurface was designed.

**Figure 6:**
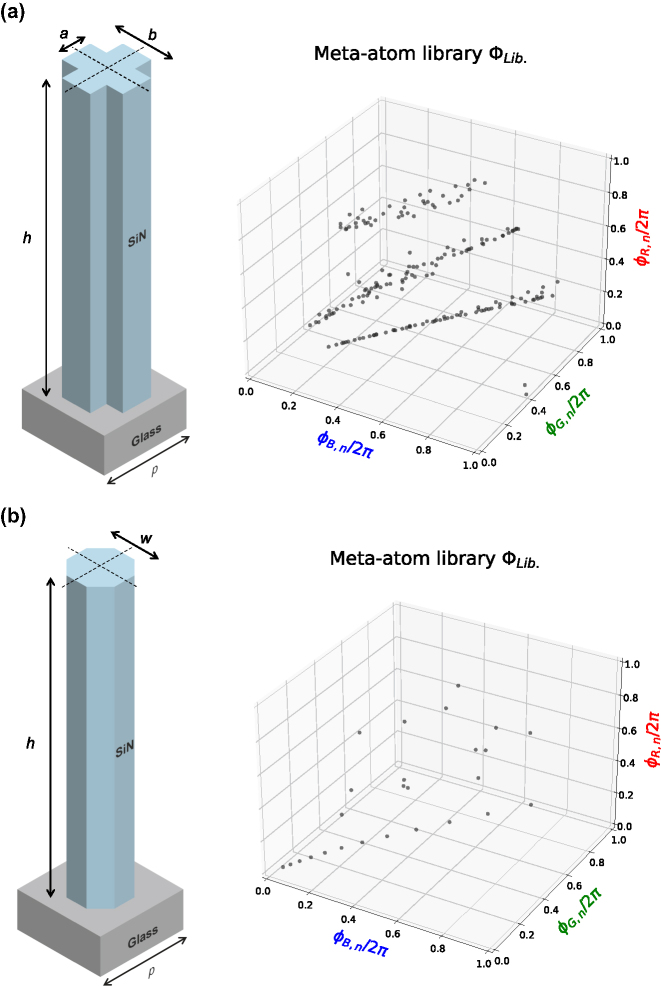
Meta-atom libraries used in this study. (a) The proposed cross-shaped SiN meta-atom with arm lengths *a*, *b* ∈ [60, 260] nm, varied in 10 nm steps. (b) The conventional octagonal SiN meta-atom with side width *w* ∈ [50, 300] nm, also varied in 10 nm steps. Both structures have a height *h* = 1750 nm and a pitch *p* = 340 nm.

To physically implement the calculated phase vectors, we designed and characterized a library of cross-shaped meta-atoms made of SiN on a glass substrate (Figure 6(a)). We performed electromagnetic simulations for operating wavelengths of 633, 532, and 445 nm, sweeping the arm lengths *a* and *b* from 60 to 260 nm in 10 nm steps, while keeping the height *h* constant at 1750 nm. The library was populated exclusively with meta-atoms exhibiting a simulated transmittance of 80 % or higher for all three wavelengths.

**Figure 7:**
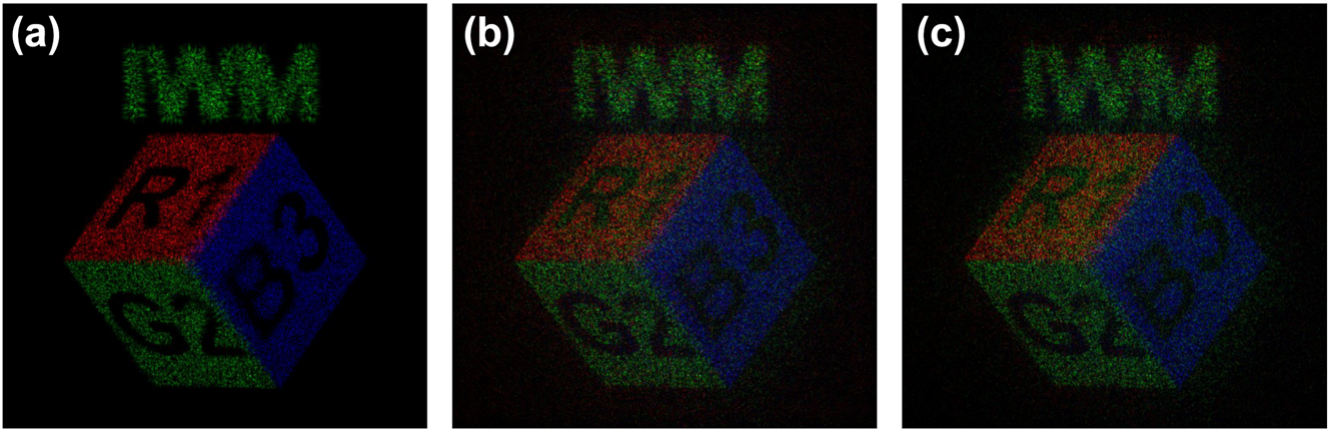
Simulated comparison of meta-atom library performance. (a) Target image. (b) Simulation image by octagonal meta-atom library. PSNR = 35.26 dB. (c) Simulation image by cross meta-atom library. PSNR = 35.67 dB. Simulation parameters: 4,096 × 4,096 pixels, 340 nm pitch, 1.0 mm target outer diameter, and 10 mm reconstruction distance, without spatial division of target 3D images. Note: Image brightness and contrast were adjusted for visualization after PSNR calculation.

To validate the superiority of our proposed structure, we compared its performance against a conventional octagonal meta-atom library based on the character projection (CP) method (Figure 6(b)). In the simulation, the amplitude of the reconstructed field was normalized, and the peak signal-to-noise ratio (PSNR) was calculated based on the resulting intensity distribution. As shown in Figure 7, simulations confirm that our cross-shaped meta-atom library achieves a higher PSNR for the reconstructed full-color 3D image. This enhanced performance is attributed to the complex geometry of the cross shape, which supports a richer set of propagation modes, enabling a more faithful reproduction of diverse phase vectors.

Finally, the complete meta-atom array was designed by matching the ideal phase vector at each pixel with the optimal meta-atom from our library. The layout of the final metasurface hologram was then generated using the gdstk library. The average transmittances of the cross-shaped meta-atom library at the RGB wavelengths were 97.5 %, 97.2 %, and 93.9 %, respectively. As these values are based on an input port set inside the glass substrate, if we consider the Fresnel reflection at the air-glass interface, the overall transmittances are calculated to be 93.6, 93.3, and 90.1 %, respectively.

## 4 Fabrication

The metasurface hologram was fabricated using the following six-step process.

1. A 1750-nm-thick silicon nitride (SiN) layer was deposited on a 20 mm square quartz glass substrate (725 μm thickness) by sputter deposition.
2. A positive-tone electron beam resist (ZEP520A-7, Zeon Co., Japan) followed by an anti-static polymer (Espacer 300Z, Resonac Co., Japan) were spin-coated onto the substrate.
3. The hologram pattern was directly written using a high-precision electron beam lithography system (F7000S-VD02, Advantest Co., Japan) with a variable shaped beam (VSB).
4. A 50-nm-thick Cr film was formed by vacuum evaporation, and a lift-off process using dimethylacetamide and a stripper (ST-120, Tokyo Ohka Co., Japan) was performed to create a chromium hard mask.
5. The SiN layer was etched using an inductively coupled plasma (ICP) reactive-ion etching (RIE) system (NE-550, Ulvac Co., Japan).
6. The residual chromium mask was removed by wet etching for 7 min in a commercial etchant (Cr-201, Kanto Chemical Co., Japan).

**Figure 8:**
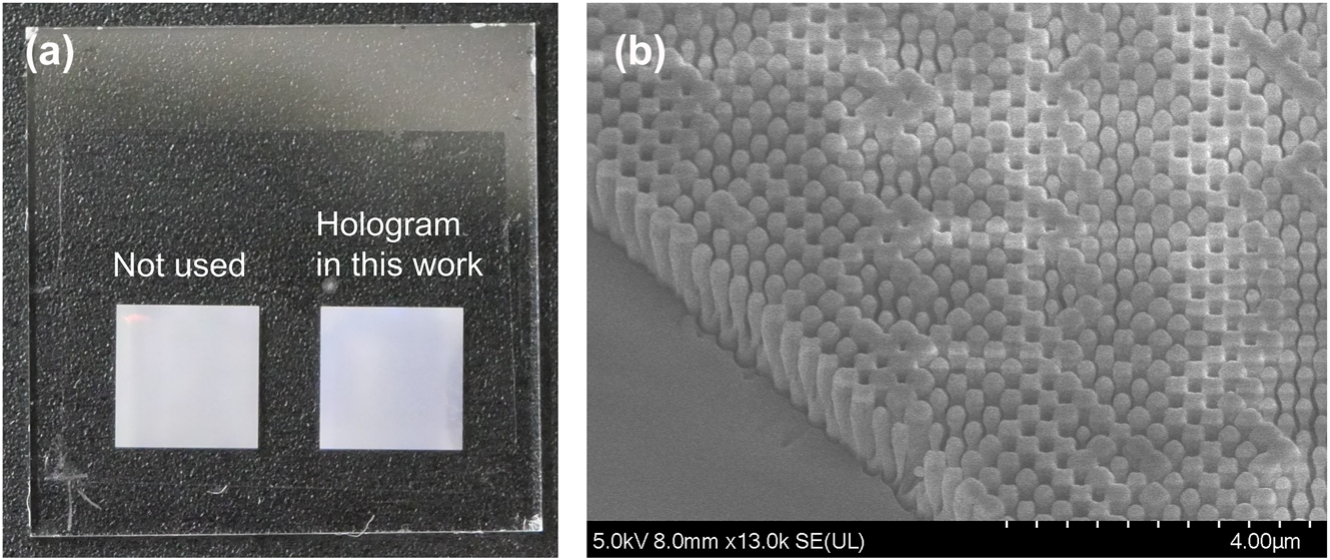
Images of the fabricated metasurface hologram. (a) Photograph of the final 5.6 mm × 5.6 mm metasurface hologram on a quartz substrate. (b) Tilted-view scanning electron microscope (SEM) image.

Figure 8 shows a photograph of the final fabricated device and a representative scanning electron microscope (SEM) image of the nanostructures. The SEM image confirms that vertically etched, high-aspect-ratio cross-shaped meta-atoms were successfully fabricated according to the design.

## 5 Results and discussion

**Figure 9:**
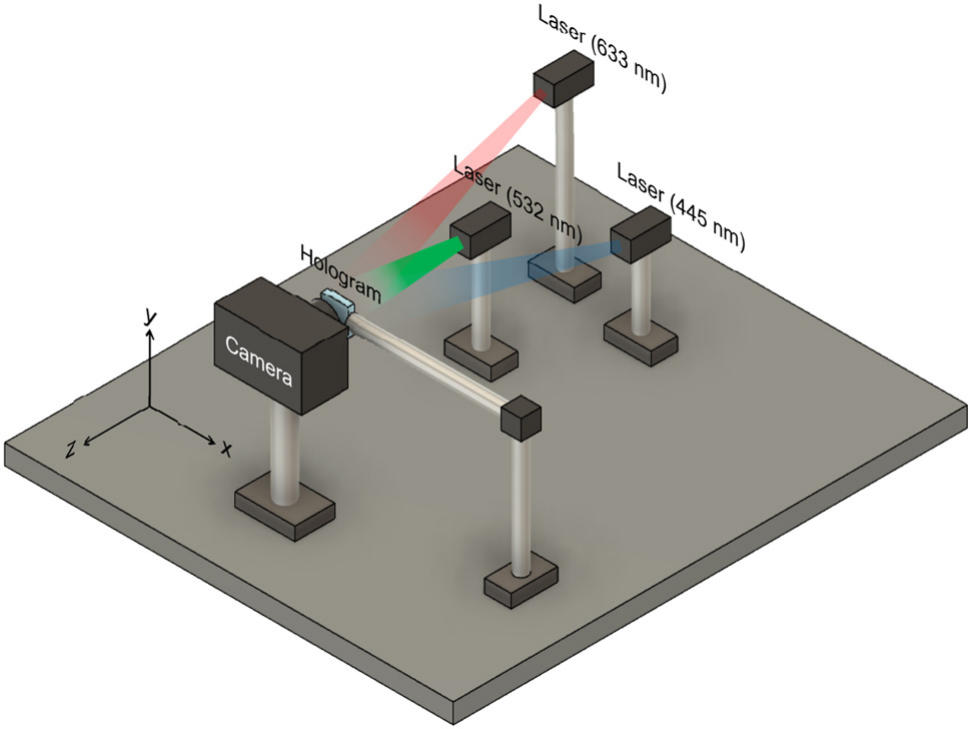
Optical setup for full-color holographic reconstruction. In our coordinate system, with the hologram at the origin (0, 0, 0), the incident positions (*x*, *y*, *z*) for the red, green, and blue lasers were set to approximately (−9.0, 4.5, −43.0), (−5.0, −5.0, −26.5), and (5.0, −7.5, −40.5), respectively (all units in cm).

The optical performance of the fabricated metasurface hologram was evaluated using the experimental setup as shown in Figure 9. Laser beams at wavelengths of 633 nm (Red), 532 nm (Green), and 445 nm (Blue) were used for reconstruction. The incident angle of each laser was precisely adjusted to ensure that the reconstructed 3D images for each wavelength were superimposed at the target location. The reconstructed image is large enough to be observed with the naked eye, and was captured using a mirrorless camera (Lumix DC-G99, Panasonic Co., Japan) with a macro lens (Lumix G Macro 30 mm/F2.8 ASPH, Panasonic Co., Japan) with the magnification of 1.52×.

**Figure 10:**
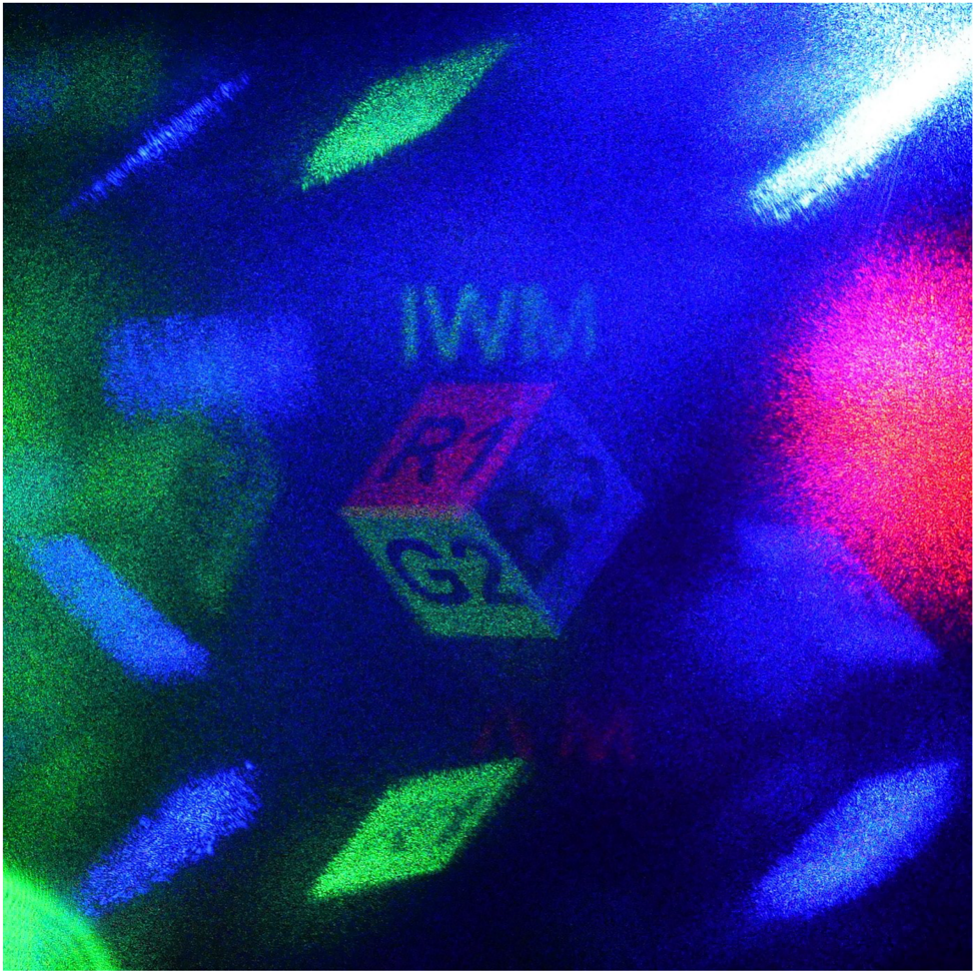
Experimentally reconstructed full-color 3D hologram. The captured image shows high fidelity to the target design while avoiding crosstalk images, demonstrating the effectiveness of our design principles.

Figure 10 shows the experimentally captured full-color holographic 3D image, which serves as a direct validation of our core design principles. Firstly, the reconstructed 3D image, which is free from overlap with crosstalk images, experimentally demonstrates the effectiveness of our spatial division strategy. Secondly, the agreement between the reconstructed 3D image and the target design confirms the accuracy of our angle correction based on Rodrigues’ rotation formula, successfully forming a superimposed full-color 3D image.

**Figure 11:**
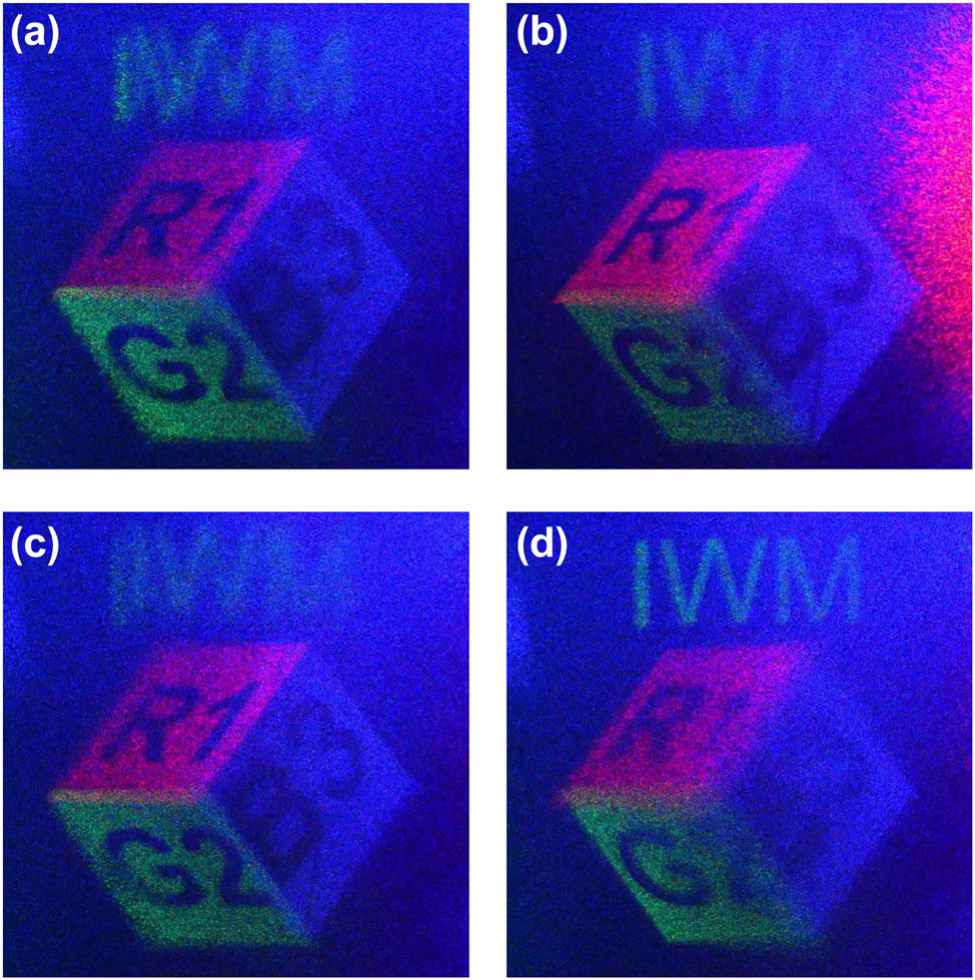
Experimental verification of the 3D properties of the reconstructed image. (a), (b) Motion parallax is confirmed by capturing the image from left (a) and right (b) viewpoints. (c), (d) Depth perception is verified by refocusing the camera between the foreground cube (c) and the background text (d).

The 3D nature of the reconstructed image was further verified, as shown in Figure 11. Motion parallax was observed by capturing the image from two different perspectives; the relative positions of the foreground cube and the background text shift as expected when viewed from the left (Figure 11(a)) and the right (Figure 11(b)). Furthermore, the depth of the 3D image was confirmed by refocusing the imaging lens. The foreground cube is in sharp focus while the background text is blurred in Figure 11(c), whereas the background text is in focus while the cube is blurred in Figure 11(d).

The measured transmittance for the RGB wavelengths were 63.4 %, 63.5 %, and 51.8 %, respectively. This is smaller than the simulated transmittance of 93.6 %, 93.3 %, and 90.1 % for each RGB wavelength and is considered to be due to fabrication error.

Thus, we have successfully demonstrated a full-color 3D metasurface hologram that simultaneously and independently controls RGB wavelengths using a single, polarization-independent meta-atom. A summary comparison of our work with key previous studies is presented in Table 1.

**Table 1:** Comparison of our work with representative full-color metasurface holograms.

Method	Pitch	Pol.	Effic. (R, G, B)
Segmented [39]	1,600 nm	Dependent	7.3 %, 19 %, 22 %
Interleaved [40]	420 nm	Dependent	Around 50 %
This work	340 nm	Independent	63.4 %, 63.5 %, 51.8 %

## 6 Conclusions

In this study, we have successfully demonstrated a full-color 3D metasurface hologram based on a single, polarization-independent meta-atom capable of simultaneous and independent phase control over three primary wavelengths.

The high-fidelity reconstruction of the full-color 3D image was enabled by a synergy of three key innovations: (1) the faithful reproduction of the target phase vector, made possible by our high-performance, silicon nitride-based cross-shaped meta-atom library; (2) the deterministic elimination of crosstalk via spatial division of target images; and (3) the precise superimposing of the color components, achieved through accurate angle correction based on Rodrigues’ rotation formula.

Our work fundamentally surpasses the critical limitations concerning pixel density and polarization dependence that have constrained previous wavelength-multiplexed metasurface holograms. This research therefore contributes significantly to the enhancement of information capacity in metasurface holography and provides a robust and practical pathway toward the realization of high-performance, full-color holographic displays for future applications.
